# Production of Gamma Alumina Using Plasma-Treated Aluminum and Water Reaction Byproducts

**DOI:** 10.3390/ma13061300

**Published:** 2020-03-13

**Authors:** Marius Urbonavicius, Sarunas Varnagiris, Liudas Pranevicius, Darius Milcius

**Affiliations:** 1 Center for Hydrogen Energy Technologies, Lithuanian Energy Institute, 3 Breslaujos, 44403 Kaunas, Lithuania; sarunas.varnagiris@lei.lt; 2 Department of Physics, Vytautas magnus University, Vileikos g. 8-304, 44404 Kaunas, Lithuania; liudas.pranevicius@vdu.lt

**Keywords:** gamma alumina, boehmite, surface area, aluminum/water reaction, hydrogen production

## Abstract

High purity hydrogen and solid-state byproducts are produced using a proposed plasma-activated aluminum and water reactions approach. These byproducts could be transformed into pure gamma Al_2_O_3_ powder material, while hydrogen can be used for electricity generation. Various chemical methods can be used for the synthesis of gamma alumina, but most could result in high levels of remaining impurities. Boehmite is a cost-effective starting material for the production of high-purity Al_2_O_3_. Herein, we present a novel method for the synthesis of boehmite and its transformation into high-specific-surface-area γ-alumina. Specifically, this method implicates the direct reaction between distilled water and plasma-treated aluminum powder. The results show the structural and morphological changes of the byproduct of the aluminum/water reaction to boehmite and γ-Al_2_O_3_ after a simple heating procedure (at 280 and 500 °C respectively). The high-purity hydrogen produced during the aluminum/water reaction can be used for the high-efficiency and environmentally friendly production of electrical energy.

## 1. Introduction

Aluminum oxide is a polymorphic material that exists in various metastable phases (γ, η, θ, χ, δ, and κ) and one thermodynamically stable α phase: alumina [1]. All metastable polymorphic phases undergo transformation steps under sufficient thermal treatment. Gamma alumina is of particular interest because of its favorable properties, such as its crystalline nature; large specific surface area and high chemical and thermal stability (important for membranes); porous morphology for the better dispersion of catalyst species; low toxicity; and acid/base characteristics, which increase (catalytic activity) the amount of adsorption sites on the surface [2,3,4,5]. γ-Al_2_O_3_ has many industrial applications, especially in the petroleum industry [6,7], automotive emission control [8], biodiesel production [9], optoelectronics [10,11], water treatment [12], membranes [13], and the improvement of wear behavior [14]. Such a polymorphic structure is considered to be a defective structure with coordinatively unsaturated aluminum cations and oxygen anions acting as acidic and basic sites, respectively [1,5]. The nature of the active surface sites depends on the positions of aluminum atoms that are not fully occupied (_V2+2/3_Al_21+1/3_O_32_, where V represents the vacancies per unit cube) [3,15]. The distribution of vacancies is related to the synthesis conditions and precursor properties [3]. Cationic vacancies (Al^3+^) are believed to be anchoring sites that bind metal particles or clusters and maintain sufficient dispersion [15].

The properties of gamma alumina are determined by the texture and morphology of the precursor and the preparation method [16]. There are various routes for the synthesis of γ-Al_2_O_3_ including the sol-gel method, hydrothermal/solvothermal processing, the precipitation (in ethanol) method, spray pyrolysis, laser ablation, a plasma jet mixed with a vapor phase precursor, and the biomimetic method using a broadleaf as a template and a nano casting route [17,18,19,20,21,22,23]. Aluminum nitrate, aluminum chloride, and alkoxides (e.g., aluminum isopropoxide) are common precursors for synthesis in aqueous or semi aqueous solutions [19,24]. Copolymers or surfactants are employed as structure-directing templates [25].

However, some of these raw materials are expensive, corrosive, and toxic chemical compounds, increasing the cost and instability of the production process [24,26]. Additionally, the aforementioned synthesis methods are often time and energy-consuming, as well as complex, involving multiple steps [20,27]. Moreover, the final γ-Al_2_O_3_ product contains impurities, such as Fe_2_O_3_, Na_2_O, SiO_2_, CaO, Cl, and sulphate compounds [28]. The concentration of impurities must be as low as possible for obtaining a highly catalytic product.

We demonstrate a proof of concept of an original method for the production of gamma alumina with a specific surface area larger than 200 m^2^/g and a crystallite size of 3–10 nm. Generally, the aluminum metal and water reaction at room temperature can be described as

2Al + 6H_2_O = 2Al(OH)_3_ + 3H_2_. (1)

Usually, the aluminum/water reaction at room temperature and reaction kinetics are mainly controlled and prevented by a thin (5–10 nm) coherent adhering layer of aluminum oxide, Al_2_O_3_, on the aluminum surface. Thus, the key for inducing and maintaining the reaction of aluminum with water at room temperature is the removal and/or disruption of the hydrated alumina layer [29]. The initial aluminum particle surfaces were modified using low-temperature hydrogen plasma. Various processes can occur during the interaction of hydrogen plasma with the aluminum particle surface: the removal of surface contamination, the formation of an altered layer by the implantation of hydrogen ions in the near the surface, the adsorption of hydrogen atoms on the surface, the formation of polar groups on the surface, the changes in the surface topography on the nanoscale, etc. In our case, hydrophobic aluminum surface was transformed into a hydrophilic surface as water contact angle decreased about three times, and the aluminum particles sank into the water immediately after immersion. This led to a fast aluminum/water reaction at 40 °C and the production of hydrogen and a solid reaction byproduct. Our previous paper describes aluminum activation in plasma, where according to the X-ray photoelectron spectroscopy results, an aluminum surface was altered, as well as kinetics of a reaction with water was described in a detailed way [30]. High-purity hydrogen can be used for electricity generation employing proton-exchange membrane fuel cells. The solid byproduct of the reaction is aluminum hydroxide, which is used as a precursor for boehmite synthesis. The purity of produced boehmite and gamma alumina depends on the primary aluminum powder, the gasses used during plasma-activation and distilled water used for reactions. Therefore, the observed level of impurities of prepared gamma alumina was insignificant. Although this paper shows novel production method of gamma alumina at the laboratory scale, but the system has a potential to be scalable and affordable production of boehmite and gamma alumina (2 kg of gamma alumina can be produced using 1 kg of aluminum) is possible especially for the sectors where only pure gamma alumina is required because of the inexpensive materials used in our technological approach, and the hydrogen produced which can be used for electricity generation.

## 2. Experimental Details

### 2.1. Synthesis Procedure

**Figure 1:**
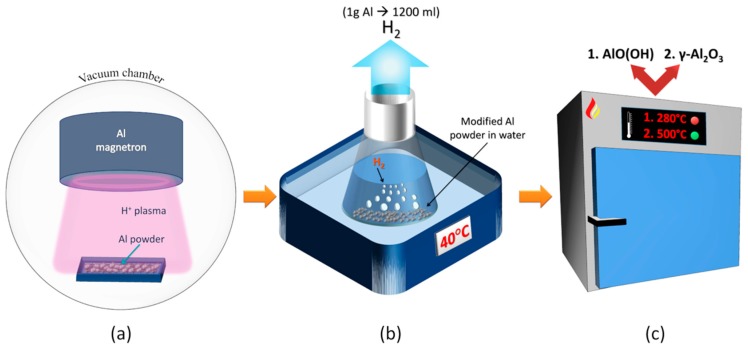
(**a**) Surface of the aluminum powder was modified under hydrogen plasma, with a magnetron as the plasma source. (**b**) Pure hydrogen and a mixture of boehmite and aluminum hydroxide were produced after the complete reaction between the modified aluminum powder and heated water. (**c**) Boehmite and gamma alumina were produced after the initial byproduct of the aluminum/water reaction was heated to 280 and 500 °C, respectively.

The modification of aluminum powder (purity 99.9%) was performed using low-temperature hydrogen gas plasma in order to overcome the thin aluminum-oxide barrier (power during activation—250 W, time—60 min). A circular magnetron (Kurt J. Lesker, Clairton, Pennsylvania, USA) with a target diameter of 95 mm was installed in the vacuum chamber and used for plasma generation (Figure 1a). A vacuum system enabled an initial pressure of 1.5 × 10^−3^ Pa. The pressure during plasma treatment was 13 Pa [30]. Modified aluminum powder was immersed into the open glass vessel with distilled water, heated to 40 °C. Although the reaction between aluminum and water is exothermic (enthalpy is about 280 kJ/mol H_2_), even better reaction kinetics can be achieved using warm water. A stirring water bath was used to control and maintain the stable temperature for 5 h which is enough for completed reaction (Figure 1b). Hydrogen gas and a solid byproduct were obtained via the reaction between plasma activated aluminum powder and water. In this case, 1 g of modified aluminum powder generated about 1200 mL of hydrogen (theoretical value 1245 mL). However, hydrogen gas was not collected or stored during this experiment and the research was orientated on the synthesis of gamma alumina. The fully reacted aluminum was transformed into a mixture of boehmite (AlO(OH)) and bayerite (Al(OH)_3_). Prior to the annealing process, the drying of the reaction byproduct was performed under ambient conditions. The pure gamma alumina preparation was implemented using a laboratory oven (SNOL 7/1300L, Utena, Lithuania). The annealing process was performed for both samples at 280 °C and 500 °C for 20 h. A heating rate of 3 °C/min was set to achieve the annealing temperature (Figure 1c).

### 2.2. Characterization

The morphology of the powder surface was evaluated using scanning electron microscopy (SEM, Hitachi S-3400N, Tokyo, Japan) at an accelerating voltage of 5 kV. The elemental compositions of the precursor sample and calcined materials were determined using energy-dispersive X-ray spectroscopy (EDS, Bruker Quad 5040, Berlin, Germany). The crystal structure was analyzed via X-ray diffraction (XRD) analysis using a diffractometer (Bruker D8, 40 kV, 40 mA, Berlin, Germany) operated in the θ–θ configuration. The main parameters of the measurements were as follows: Cu Kα radiation (λ = 0.15406 nm), a scan range of 25–70°, a 0.3° fixed div slit. The mean crystallite size was estimated using the TOPAS software (version 4.1) (Lorentzian convolution was applied). X-ray photoelectron spectroscopy (XPS, PHI 5000 Versaprobe, Chanhassen, Minnesota, USA) was performed to determine the elemental composition of the surface. Quantitative analysis of the XPS spectra was performed using the software Multipak. Spectra were recorded with a pass energy of 187.85 eV. The main XPS measurement parameters were as follows: monochromated 1486.6 eV Al Kα radiation, 25 W beam power, 100 μm beam size, and 45° measurement angle. Brunauer–Emmett–Teller (BET) surface-area analysis was performed using a nitrogen adsorption–desorption isotherm obtained via a sorptometer (Kelvin-1042, Talinn, Estonia). All the samples were degassed at 100 °C for 2 h prior to the BET measurements. The pore size distribution of gamma aluminum oxide was calculated from desorption branch of the isotherm by the Barrett-Joyner-Halenda (BJH) method.

## 3. Results and Discussion

**Figure 2:**
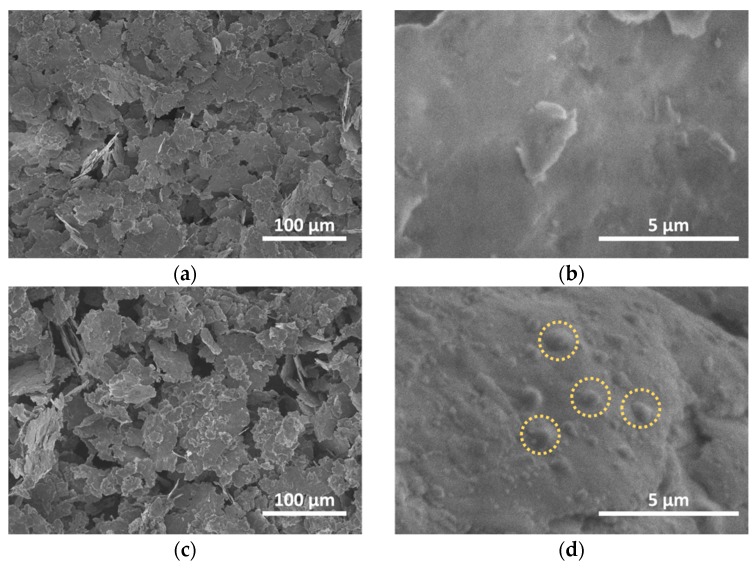
(**a**–**b**) SEM images of untreated Al powder and (**c**–**d**) H_2_ plasma treated at 250 W for 60 min.

SEM images of untreated aluminum powder and treated under hydrogen gas plasma are presented in Figure 2. Powder contains of irregularly-shaped Al flakes that size varies from 10 up to 75 µm. The surface of untreated Al powder seems to be relatively smooth (Figure 2b), while some areas of plasma treated surface revealed irregularities, cracks and bubbles (Figure 2d). The results of TRIM computer code [31], based on Monte Carlo simulations, showed that the 1 keV H^+^ ions can perpetrate into approximately 20 nm depth of Al material. The native oxide barrier was passed by the incident ions, which is about 5 nm and stopped in the Al flakes near the surface region. In the presence of the barrier Al_2_O_3_ layer on the surface, the released hydrogen from the bulk is accumulated in the form of bubbles, which are located on the flakes surface. XRD analysis confirmed that no aluminum hydride was formed during plasma activation processes. The insignificant increase of BET surface area was measured after plasma activation (3.75 m^2^/g), while the value for initial Al powder was 3.51 m^2^/g. A detailed interpretation of Al powder surface morphology alteration and particularly change of surface chemical structure caused by plasma treatment was presented in our previous work [30].

**Figure 3:**
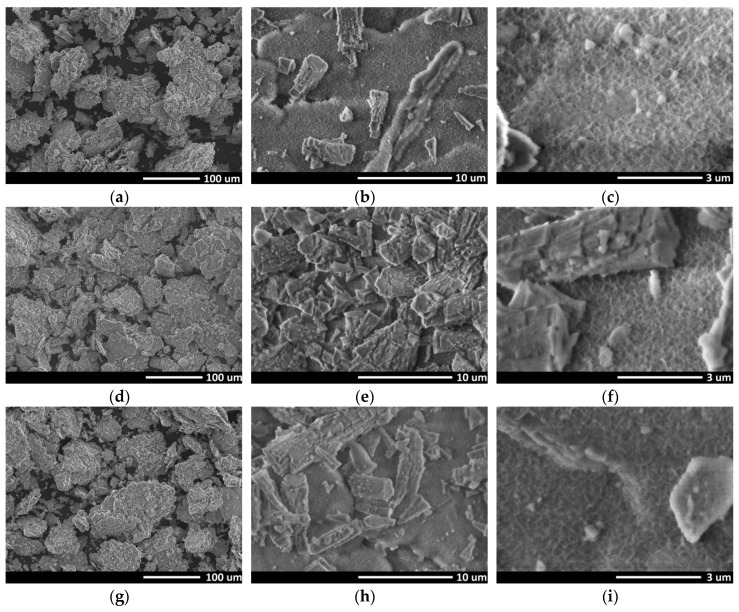
SEM images: (**a**–**c**) initial byproduct after the aluminum/water reaction (mainly Al(OH)_3_), (**d**–**f**) the boehmite (AlO(OH)) after aluminum hydroxide heated at 280 °C, (**g**–**i**) the gamma alumina after aluminum hydroxide heated at 500 °C.

SEM was used for the surface morphology analysis of the byproduct (Figure 3a–c) obtained after the aluminum/water reaction, the boehmite (Figure 3d–f), and the gamma alumina (Figure 3g–i) synthesized using heat pretreatment. Results showed that the surface remains relatively homogeneous despite the transformations from byproduct of aluminum/water reaction into the boehmite and γ-Al_2_O_3_ phases. Figure 3a,d,g revealed that larger, irregularly-shaped aggregates with sizes varying from several to tens of microns were formed due to agglomerations between the smaller powder particles. The enlarged images (Figure 3c,f,i) showed porous surfaces with self-connected pores in all cases.

The EDS results, which are summarized in Table 1, confirmed that all samples consist of oxygen, aluminum and negligible amount of carbon only. The carbon content is related to the uptake of environmental gases that occurred during the transfer of the sample into the atmosphere. The analysis of oxygen/aluminum ratio revealed decreased tendencies (from 3.36 to 2.05 for aluminum/water byproduct and gamma alumina, respectively). This process was invoked by dihydroxylation through the heat pretreatment.

**Figure 4:**
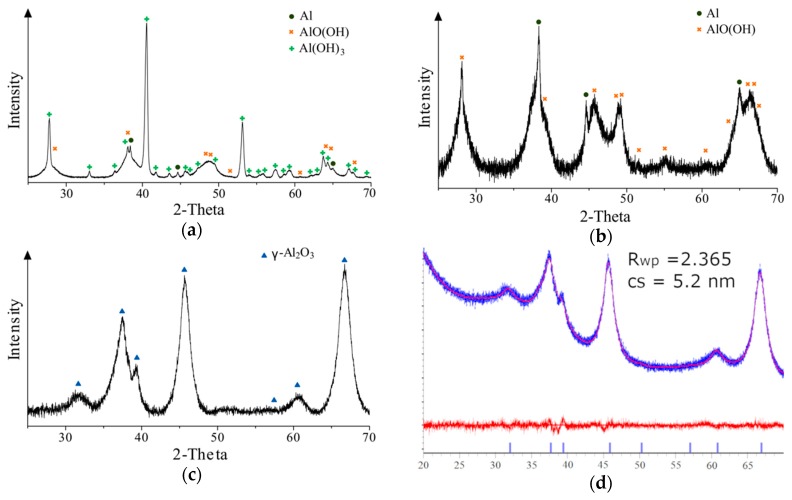
XRD results: (**a**) the byproduct after aluminum/water reaction, (**b**) aluminum hydroxide transformed into boehmite after heating at 280 °C, (**c**) gamma alumina after heating at 500 °C and (**d**) crystallite size calculation of gamma alumina using Topas software.

The XRD technique was used for the analysis of byproduct and the phase transformation after heat treatment process. The results showed that the byproduct consists of aluminum hydroxide (Al(OH)_3_), boehmite (AlO(OH)) and negligible part of pure aluminum after the aluminum/water reaction (Figure 4a). It was observed that the predominant compound was monoclinic Al(OH)_3_ (space group symmetry P21/a). The indicated crystallite size was 59.7 nm. The aluminum peaks were detected due to imperfect exothermic reaction between plasma-activated aluminum and water. Presumably, the byproduct produced during the Al-water reaction later acts as a barrier for water molecules that leads to a decrease of reaction kinetics.

Boehmite with an orthorhombic unit cell (space group symmetry Cmcm) was obtained after the heat pretreatment of the initial byproduct near its melting-point temperature (Figure 4b). The broadness of the peaks indicates the nanoscale size of the crystallites. The average crystallite size was 3.5 nm. A small amount of aluminum was present in the phase composition. Boehmite can be used as an additive for the production of other materials, such as ceramics, surface coatings, and pharmaceuticals [32].

The synthesis of gamma alumina was performed using boehmite, which was thermally treated at 500 °C (Figure 4c). This process induced the formation of γ-Al_2_O_3_, which distinguished spinel-like face-centered cubic structure (space group symmetry Fd-3m) due to the structural water loss and surface hydroxyl groups desorption. It was noted that with the synthesis of new phase, the aluminum and boehmite peaks fully disappeared. The observed XRD pattern consisted of three well-defined and few small γ-Al_2_O_3_ diffraction peaks at 31.7°, 37.5°, 39.2°, 45.7°, 60.6°, and 66.7°. These peaks can be attributed to the (220), (311), (222), (400), (511), and (440) orientations, respectively. The synthesis of nanocrystalline product was approved by calculation of crystallite size (calculated by Topas which applied Lorentzian convolution—5.2 nm, R_wp_ = 2.365), as well as the occurrence of broadened peaks (Figure 4d). The peak broadening can be related to the formation of defects. Moreover, many other authors declared that the crystallite size of gamma alumina tends to be less than 10 nm [33,34,35,36]. There were no additional peaks, which could correspond with new compounds, based on XRD analysis. This indicated that high purity crystalline gamma alumina was synthesized.

**Figure 5:**
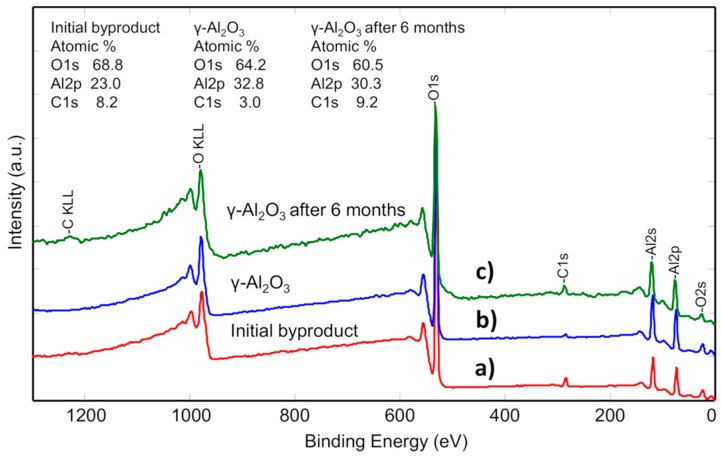
Results of XPS elemental composition: (**a**) byproduct after aluminum/water reaction, (**b**) the gamma alumina after heat-treatment at 500 °C for 20 h and (**c**) gamma Al_2_O_3_ stored for 6 months under the atmospheric conditions.

The analysis of XPS spectra (Figure 5) showed that the same three components (carbon (C1s), oxygen (O1s) and aluminum (Al2p)) were observed for both initial powders and the gamma alumina. Analysis depth of EDS technique (Table 1) ranges from 1 up to 2 micrometers while XPS is a surface sensitive measurement method which can detect chemical elements at the very top surface up to 10 nm depth. Accordingly, the nanoscale analysis did not indicate any additional impurities except carbon (the measurement area diameter—100 µm). The presents of carbon contamination on the very surface could be explained by the fact that all samples were kept in atmospheric conditions before and after treatment. As surfaces of all the samples were active, it could easily adsorb carbonaceous species from the atmosphere. Despite that prepared gamma Al_2_O_3_ was stored in the closed glass vessel for 6 months, XPS showed that carbon concentration increased about 3 times (Figure 5c), confirming presumptions about high surface activity for adsorption of contaminants. The amount of oxygen diminished from 68.8 to 64.9 at.%. Contrarily, the aluminum concentration increased from 23 at.% of initial byproduct to 32.8 at.% and 30.3 at.% of as-prepared and stored for 6 months gamma alumina, respectively. The aluminum deficiency was noticed by analyzing the gamma alumina surface, while the Al/O ratio was fixed at 0.51 (the theoretical ratio of Al/O = 0.66). We presume that the occurrence of aluminum deficiency can be related to vacancies formation of cationic Al^3+^ (see introduction).

BET surface area is recognized as an essential characteristic for high catalyst activity. The specific surface area depends on the number of coordinatively unsaturated sites. The dispersion of active catalytic species can be performed on such areas. The increment of specific surface area was observed by comparing a byproduct of aluminum/water reaction (206.8 m^2^g^−1^) and the boehmite powder (268.1 m^2^g^−1^). However, a slight decrease of specific surface area (247.9 m^2^g^−1^) was observed after the synthesis of gamma alumina. Such a decrease can be related to the changes of crystallite size (XRD results in Figure 4).

Presumably, the presence of uncoordinated surface sites and large BET surface area led to the agglomeration of powder particles (SEM views). Nevertheless, high purity and large specific surface area nanocrystalline gamma alumina was prepared by plasma activated aluminum and water reaction.

**Figure 6:**
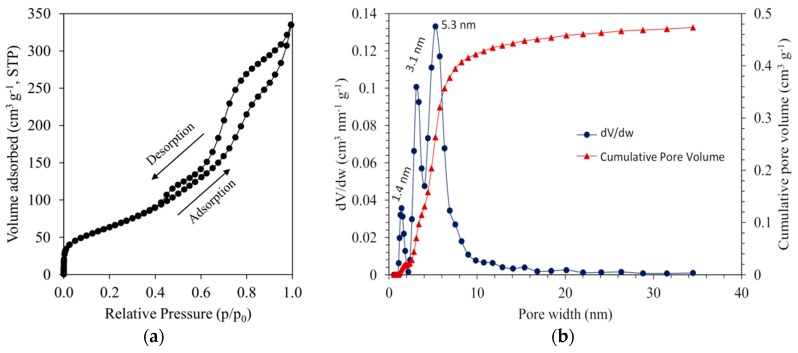
(**a**) N_2_ adsorption-desorption isotherms and (**b**) differential (blue curve) and cumulative (red curve) pore size distribution of gamma aluminium oxide.

The adsorption-desorption isotherms of N_2_ and pore size distribution curves (differential and cumulative) of synthesized gamma aluminum oxide are illustrated in Figure 6a,b, respectively. According to the IUPAC classification, gamma alumina exhibits typical IV isotherm hysteresis loop (between type H1 and H2) at relative pressure range of *p/p*_0_ = 0.4–0.97, indicating a mesoporous structure (Figure 6a). The observed sharp slope at very low *p/p*_0_ probably is due to an additional micro-porosity of the sample (although the volume is small). This type of isotherm is suggesting that material may have complex interconnected pores [37]. As well, the H1 type of isotherm is associated with the capillary condensation in pore channels with possible channel adjustment [38].

The analysis of pore size distribution (Figure 6b) shows three narrow distributions that are centered around at 1.4, 3.1 and 5.3 nm, respectively. Material with pore sizes in the range between 2 and 50 nm is considered to be a mesoporous material which is in agreement with isotherm (Figure 6a).

The gamma Al_2_O_3_ synthesis methods presented by other authors are reviewed in Table 2. It could be seen that the main methods used for gamma Al_2_O_3_ production are chemical-based technologies (precipitation, sol-gel), while one of the most used precursors is aluminum isopropoxide. Normally, the impurities can be found in gamma alumina, which was synthesized by chemical-based technologies. For instance, Fe_2_O_3_, Na_2_O, SiO_2_, and CaO impurities were found by using the precipitation method with sodium aluminate precursor [39]. Impurities were found by Ali et al. as well, which used precipitation with AlCl_3_ × 6H_2_O precursor [40]. Unfortunately, some works present lack of information about the elemental composition, which in turn invokes uncertainty about purity of synthesized gamma alumina. The technique used in this work presents the possibility to produce gamma Al_2_O_3_ from pure aluminum/water reaction byproduct (boehmite) avoiding any chemicals, which ensures high purity level of synthesized gamma alumina. Also, our technique led to producing gamma alumina with high specific surface area (247.9 m^2^/g) and pore characteristics, which are in average value compared to other methods. Moreover, the technique presented in this work is non-hazardous and environmentally-friendly with just a few steps (plasma treatment, aluminum/water reaction, thermal treatment). It does not require any complicated or expensive equipment nor chemicals, which are commonly used in other techniques. In terms of cost efficiency, the price of gamma Al_2_O_3_ synthesized by presented technique possibly can be lower compared to some well-known suppliers, which offer gamma alumina with similar characteristics (very pure γ-Al_2_O_3_ can cost up to several hundred euros).

## 4. Conclusions

We demonstrated the synthesis of γ-Al_2_O_3_ with a specific surface area of 247.9 m^2^g^−1^ from boehmite (AlO(OH)) produced using water/plasma-activated aluminum reaction byproducts. The hydrogen plasma activation of initial Al flakes leads to hydrogen ions implantation in near surface region with further accommodation of hydrogen into bubble like structures on the Al surface. These surface irregularities could initiate formation of the cracks in a natural aluminum oxide layer and help for the water to penetrate into depths of materials and react with pure aluminum. The purity of synthesized gamma alumina mainly depended on the purity of working gases used during the plasma activation and the purity of primary aluminum powder. Irregularly-shaped metallic aluminum powder with a flake-like structure whose size varies from 10 up to 75 µm was used. The received γ-Al_2_O_3_ is without traces of Fe_2_O_3_, Na_2_O, SiO_2_, CaO, Cl, and sulphate compounds contamination which was produced as irregularly-shaped aggregates of smaller powder particles. The size of aggregates varied from several to tens of microns. However, received γ-Al_2_O_3_ is very active and could easily absorb carbon-based contaminants if kept in atmospheric conditions even after keeping it for more than 6 months in atmospheric conditions.

## 5. Patents

Discussed methods for the production of pure gamma aluminum oxide, as well as hydrogen production from water has become the subject of PCT application called a “Method for synthesis of γ-aluminium oxide using plasma-modified aluminium and water reaction”. Patent application was published on October 4, 2019 (No. WO2019186234).

## Figures and Tables

**Table 1:** Chemical composition of the precursor (Al(OH)_3_) and calcined materials.

Samples	O, at.%	Al, at.%	C, at.%
Initial byproduct	75.71	22.52	1.77
Boehmite	67.26	29.77	2.97
Gamma alumina	64.82	31.53	3.66

**Table 2:** Gamma Al_2_O_3_ synthesis methods and its main characteristics.

Method	Precursor	BET Surface Area (m^2^/g)	Pore Characteristics		Ref.
			Volume (cm^3^/g)	Diameter (nm)	
Lime-sinter	Fly ash	196.1–404.3	0.23–0.82	2.77–15.22	[41]
Precipitation	Sodium aliuminate	269.9	0.57 (mL/g)	-	[39]
Calcination	Polyoxohydroxide aluminum	72–282	0,13–0,24	3,5–10,3	[42]
Precipitation	AlCl_3_ × 6 H_2_O	112.9	-	4.13	[40]
Sol-gel	Aluminate liquor	138.8–201.1	0.16–0.24	4.7–7.8	[43]
Two-step pyrolysis	Al-based metal organic	240–350	0.52–0.63	7.2–8.6	[44]
Evaporation-induced self-assembly	Aluminum isopropoxide	234–285	0.23–0.49	3.8–6.7	[45]
Sol-gel	Aluminum isopropoxide	306–351	0.226–1.29	5.34–6.13	[46]
Solvent-deficient	Tri-sec-butoxide aluminum	192–375	0.44–0.80	7.4–16.2	[47]
Sol-gel	Aluminum isopropoxide	180–260	0.8–1.4	7-37	[48]
Commercial	Unknown	220–280	0.8–1.2	-	Alfa Aesar by Thermo Fisher Scientific
